# Fire, Fuel Composition and Resilience Threshold in Subalpine Ecosystem

**DOI:** 10.1371/journal.pone.0012480

**Published:** 2010-08-30

**Authors:** Olivier Blarquez, Christopher Carcaillet

**Affiliations:** 1 Paleoenvironments and Chronoecology, École Pratique des Hautes Études, Montpellier, France; 2 Centre for Bio-Archaeology and Ecology, Université Montpellier 2, Centre National de la Recherche Scientifique, Montpellier, France; University College London, United Kingdom

## Abstract

**Background:**

Forecasting the effects of global changes on high altitude ecosystems requires an understanding of the long-term relationships between biota and forcing factors to identify resilience thresholds. Fire is a crucial forcing factor: both fuel build-up from land-abandonment in European mountains, and more droughts linked to global warming are likely to increase fire risks.

**Methods:**

To assess the vegetation response to fire on a millennium time-scale, we analyzed evidence of stand-to-local vegetation dynamics derived from sedimentary plant macroremains from two subalpine lakes. Paleobotanical reconstructions at high temporal resolution, together with a fire frequency reconstruction inferred from sedimentary charcoal, were analyzed by Superposed Epoch Analysis to model plant behavior before, during and after fire events.

**Principal Findings:**

We show that fuel build-up from arolla pine (*Pinus cembra*) always precedes fires, which is immediately followed by a rapid increase of birch (*Betula* sp.), then by ericaceous species after 25–75 years, and by herbs after 50–100 years. European larch (*Larix decidua*), which is the natural co-dominant species of subalpine forests with *Pinus cembra*, is not sensitive to fire, while the abundance of *Pinus cembra* is altered within a 150-year period after fires. A long-term trend in vegetation dynamics is apparent, wherein species that abound later in succession are the functional drivers, loading the environment with fuel for fires. This system can only be functional if fires are mainly driven by external factors (e.g. climate), with the mean interval between fires being longer than the minimum time required to reach the late successional stage, here 150 years.

**Conclusion:**

Current global warming conditions which increase drought occurrences, combined with the abandonment of land in European mountain areas, creates ideal ecological conditions for the ignition and the spread of fire. A fire return interval of less than 150 years would threaten the dominant species and might override the resilience of subalpine forests.

## Introduction

Theoretical community dynamics are often analyzed over relatively short-term periods of weeks or decades, which restricts the potential to assess the mechanisms that link disturbances to biological assemblages [1], [2]. However, long-term analyses, over time-spans of centuries, are needed to decipher: the processes controlling the occurrence of disturbances; their affects on ecosystem properties; and, feedbacks to disturbance regimes [3], [4]. These long-term studies enable the effects of repetitive processes on the resilience of communities to be observed, thereby highlighting: (1) the thresholds that, if over-reached, can threaten ecosystems [5] and (2) any decrease in the supply of ecosystem services [6]. Long-term analyses of sites that have experienced different disturbance histories provide extensive information about the mechanisms of plant dynamics [7]. Post-fire dynamics are highly dependent on factors such as climate and landscape pattern – factors that are projected to change in the near future due to global warming and changes in land use [8]. We therefore stress the need for a greater understanding of fire-ecosystem relationships over time-scales of centuries, for ecosystems such as those at high altitude or latitude that are sensitive to global changes [9].

Specifically, global warming, and its projected increase in the frequency of drought, may augment the risk of fire in southern Europe [10], [11] with potential effects on subalpine forests [12]. Fuel build-up and greater connectivity between forests following land-use abandonment in these regions may also promote an increase in the likelihood of fire [13]. Thus, an understanding of the resilience threshold to fire for these ecosystems, and the linkages with fuel modification, is crucial to forecasting their response to global changes. We therefore address the following questions in our study: (1) in subalpine ecosystems, what species are likely to generate a fuel build-up and so increase fire risk? (2) What succession patterns are likely to follow a fire? Then, we aim to (3) identify a fire resilience threshold for these ecosystems.

To answer the above questions, we analyzed: (i) sedimentary plant macroremains, which allowed us to decipher community dynamics by bridging the ecology and paleoecology [14]; and (ii) sedimentary charcoal remains, in order to reconstruct the occurrence of paleofires [15]. The time-span studied covers the last 8000 years of the current interglacial period, namely the Holocene. The sediments were sampled from two subalpine lakes of the internal western Alps: Lago Perso and Lac du Loup. These lakes have small surface areas (<1500 m^2^) and are fed from limited watersheds (<0.65 km^2^), thereby offering appropriate conditions to record stand-to-local ecological processes [14]. Superposed Epoch Analysis (SEA) was used as a time series analysis to decipher the behavior of a response variable to multiple discrete particular events [16], [17]. SEA has previously been successfully used in ecology to analyze climatic influence on fire occurrences [18], [19], and in atmospheric science to decipher temperature responses to volcanic eruptions [20]. Here we used SEA to decipher the behavioral responses of the main functional subalpine species to fire over the past 8000 years, assuming that assemblages of sedimentary plant macroremains provide evidence of the local composition of plant species, and that influxes of macroremains provide information on changes in the biomass of species present in the lake surroundings. Paleoecology can provide valuable information on how ecosystem dynamics are shaped by fire over long time-scales, and the rules by which different species respond to fire.

## Results

### Cumulated macroremain influxes in relation to fire

**Figure 1:**
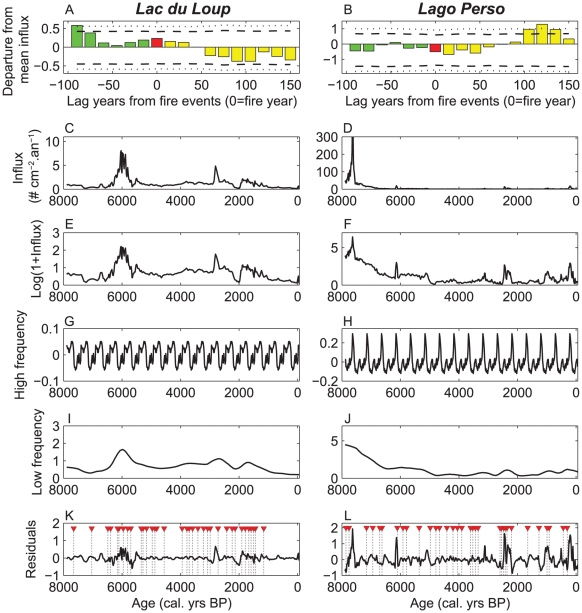
Superposed Epoch Analysis (SEA) technique outline. SEA results applied to the sum of transformed total macroremain influxes for Lac du Loup (A) and Lago Perso (B). Pre- and post-fire transformed means of macroremains influx are colored in green and yellow, respectively; times of fires are in red, with 95% and 99% confidence intervals (CI) of means given by dashed and dotted lines, respectively. (C,D) The sum of raw macroremain influxes for the two sites and, (E,F) the sum of transformed macroremain influxes (see Material and Methods for details on mathematical formula). (G,H) High and (I,J) low frequency trends in macroremain influx sum decomposed from LOESS fitting. (K,L) Residuals from LOESS decomposing used to perform SEA, where triangles (∇ in red) and dashed lines give the date of reconstructed fire events.

In total, SEA was carried out on 35 of the highest charcoal peaks related to fire events for Lago Perso and Lac du Loup during the past 8000 years. SEA of the total influx of plant macroremains showed a lower average macroremain influx following fires at Lac du Loup for the −90/+150-yr period (Table 1, Figure 1A), while at Lago Perso a significant (>99% confidence interval, CI) increase of influx is observed over the 100-year period following fires (Figures 1B). The decrease at Lac du Loup persisted for over 150 years (Figure 1A).

**Table 1:** Differences between pre- and post-fire abundance of macroremains SEA mean influx following fire events for different time windows.

	[−90–0] vs [0–150]						[−90–0] vs [0–90]					[−60–0] vs [0–60]					
	Lac du Loup			Lago Perso			Lac du Loup			Lago Perso		Lac du Loup			Lago Perso		
Taxa	p-val	signif		p-val	signif		p-val	signif		p-val	signif	p-val	signif		p-val	signif	
*Larix decidua*	**0.003**	**	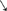	0.181			**0.026**	*	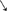	0.065	°	0.343			**0.029**	*	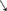
*Pinus cembra*	**0.008**	**	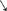	**0.031**	*	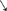	0.065	°		0.240		0.686			0.343		
*Betula* sp.	0.383			**0.020**	*	↗	0.061	°		0.087	°	**0.029**	*	↗	0.200		
Ericaceae	0.368			0.444			**0.026**	*	↗	0.426		0.057	°		0.400		
Herbs	0.274			0.545			0.418			0.589		0.086	°		0.686		
Influx sum	**0.007**	**	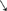	0.263			0.065	°		0.937		0.686			0.114		

Wilcoxon Mann-Whitney non-parametric test *p*-values and significant levels are given. Where differences exist, arrows show the direction of variation of macroremain influxes (** *p*<0.01, * *p*<0.05, ° *p*<0.1).

### Species behavioral response to fires

**Figure 2:**
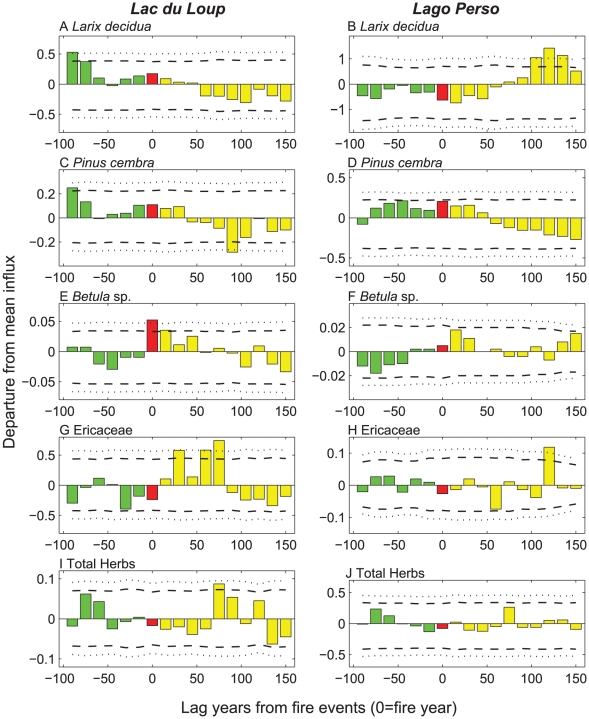
Superposed Epoch Analysis results applied to transformed macroremain influxes of main subalpine species for the two sites. Pre- and post-fire transformed macroremains influx means are colored in green and yellow respectively; fire-year transformed macroremains influx means are in red with 95% and 99% CI of means given as dashed and dotted lines, respectively.

During the “fire-year” period that uncovers 7.5 years before and after the charcoal peak (Figure 2, red bars), all species except larch, *Larix decidua* (Figure 2A,B), showed the same pattern of averaged macroremains influx at both sites. Irrespective of site, the influxes of arolla pine (*Pinus cembra*) and birch (*Betula* spp.) were always greater than the mean at the time of fire (Figure 2C–F), while the scores for ericaceous species and herbs were always lower (Figure 2G–J). The response of *Larix decidua* macroremains to fires appears to be site dependent since, during a fire-year, averaged macroremain influxes were greater than the mean for Lac du Loup (Figure 2A). In contrast, macroremain influxes were lower than the mean at Lago Perso (Figure 2B). At Lac du Loup, the SEA results showed a significantly decreasing pattern for the −90/+150-yr and the −90/+90-yr time window, while a significantly increasing pattern was apparent for Lago Perso after 100 years, with the averaged influx clearly surpassing the 95% CI (Figure 2A,B, Table 1, *p*<0.01). Within a −60/+60-yr window, no significant response could be observed at either site (Table 1), suggesting that the behavior of *Larix decidua* is independent of fire.

The relations between the averaged influxes of *Pinus cembra*, *Betula* spp., Ericaceae and herbs to fires were quite similar at both sites (Figure 1C–J). *Pinus cembra*, which is prevalent before fire, significantly declines afterwards at both sites (−90/+150-yr window, *p*<0.05; Table 1). At Lac du Loup, *Pinus cembra* is significantly more abundant 90 years before a fire, and less abundant 90 years after (averaged influx >95 and 99% CI, respectively). The −90/+90-yr and −60/+60-yr window exhibited no effect of fire events on *Pinus cembra* macroremains, regardless of the site.

Averaged influxes of *Betula* spp. rose significantly following fires at Lago Perso for the −90/+150-yr window (*p*<0.05). At Lac du Loup, the sole significant increase in *Betula* influx after fires is observed for the −60/+60-yr window (*p*<0.05), showing a very high *Betula* influx during the fire-year and continuing until 15 years after (99 and 95% CI, respectively).

A significant increase in the averaged influx of Ericaceae following fire is observed at Lac du Loup for the −90/+90-yr window (*p*<0.05, Table 1), with significant increases in average influx occurring after fires at +30 (>95% CI), +60 and +75 years (>99% CI) for Lac du Loup (Figures 2G) and +120 years for Lago Perso (>99% CI; Figure 2H).

Average influxes of total herbs showed no response to fire irrespective of site and time window. However, a significant but temporary increase in average herb influxes was detected +75 years (>95% CI) after fires for Lac du Loup (Figure 2I).

### Modeling succession patterns

**Figure 3:**
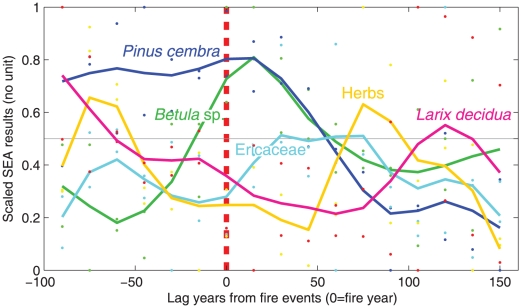
Modeled behavior of plants pre- and post-fire using Superposed Epoch Analysis summarized results for the two lakes. SEA means were transformed using min/max scaling followed by LOWESS curve fitting to highlight macroremains trajectories by species for the two lakes.

Succession pattern of subalpine species are modeled by combining SEA results from the two lakes (Figure 3). The resulting pattern shows that fires are immediately followed by an increase in *Betula* that lasts for approximately 50 years, whereas before a fire, *Betula* reaches its minimum values. This rise in *Betula* is immediately followed by an increase in ericaceous species, with maxima recorded between 25 and 75 years after fires. The maximum for herbs occurred 75–100 years after fires. The abundance of *Pinus cembra* is very high before fires, and continuously declines for a 100-years period thereafter. The pattern for *Larix decidua* shows a slight augmentation 100–150 years after fires and a progressive decline during the 100 years preceding fire.

## Discussion

The analysis of macroremains influx, which reflects plant biomass in the surrounding environment, provides a comprehensive scenario of how different species interact with long-term fuel build-up and fire occurrence. We show that elevated influxes of *Pinus cembra* macroremains always precede the occurrence of fire; an observation that argues in favor of the hypothesis that over the long-term, a fuel build-up is required for the occurrence of fires. This does not however, rule out the importance of climate as an influencing factor on the occurrence of fires [21], [22].

### Pre- and post-fire plant behavior

Superposed Epoch Analyses (SEA) from both sites demonstrated that *Pinus cembra* is the required fuel for fire to spread (Figure 2C,D). A significant response of *Pinus cembra* was only observed for the longest time window at both sites (−90/+150-yr, Table 1). The shortest time windows are not large enough to detect the responses of such long-lived species to fires. Fire-vegetation analyses based on the analysis of a single site between 9 and 5000 cal BP already suggested that *Pinus cembra* was the main fuel of subalpine fires in the area[23]. Similarly, our results, which cover a longer period (i.e. 8000 years) at two sites, support this conclusion. However, our dataset also contains *Larix decidua* as a co-dominant species; which was absent from the Genries' dataset as *Larix decidua* is not frequently found on south-facing slopes [23]. Our dataset was gathered from two sites on north-facing slopes that capture less insolation (colder) and have shorter growing season. The decrease of *Pinus cembra* after fire, but not its total disappearance, is probably linked to events where only a few trees are killed or damaged by fire due to surface fires, which tend to exclude complete stand-replacing fires [24].

SEA scores showed contrasting patterns of *Larix decidua* behavior to fires that were site-dependent (Figure 2A,B). Before fires, *Larix decidua* was abundant at Lac du Loup (Figure 2A), but not at Lago Perso (Figure 2B). At Lac du Loup, fires promoted a decrease in *Larix decidua*, while the same conditions favored the species at Lago Perso. These differences may be explained by the combination of other disturbances associated with fires, e.g. avalanches, outbreaks of insects, or up-rooting of trees [25], [26]. These may have amplified or altered the consequences of disturbance from fire alone. Today, the area surrounding Lac du Loup is sensitive to snow avalanches [27] suggesting that partial tree removal by fire may have promoted snow avalanches [28], which might have ultimately led to the apparent decrease of *Larix decidua* due to the additional time involved necessary to overcome stand resilience. In contrast, SEA results from Lago Perso show that fire clearly stimulated the abundance of *Larix decidua* after a period of 100 years (Figure 2B). It is noteworthy that snow avalanches around this site were unlikely due to unfavorable topography. However, given the absence of any clear observations involving contemporary interactions between fire and other disturbances in the subalpine Alps, and the promotion of long-lasting *Larix decidua* stands, any further hypothesizing on this subject would be speculative. Furthermore, recent dendrochronological studies of burned stands of mixed *Larix decidua*-*Pinus cembra* within the subalpine belt failed to discover fire scars on old-growth *Larix decidua* [29], suggesting that the thick bark of *Larix* prevents mature trees from suffering cambial damage due to fire [30]. In addition, because *Larix decidua* probably responds more intensely when several disturbances interact, this makes it more difficult to define an unambiguous response of *Larix decidua* to fire.

*Betula* increased in abundance shortly after fires (Table 1, −60/+60-yr windows), even though this taxon was scarce before fires (Figure 2E,F). *Betula pendula* Roth. and *B. pubescens* Ehrh. are both short-lived species most commonly found following certain disturbances, and in stressed habitats such as wetlands. Thus, the observed response of *Betula* to fires is consistent with the known ecology of birch species [31], particularly in cold conifer ecosystems [32].

Ericaceous species are greatly affected by surface fires but are nonetheless able to recover their abundance within 5–15 years [33]. Indeed, during the fire-year periods (red bars, Figure 2G,H), SEA gave negative influxes for Ericaceae. The time needed for their recovery is longer than observed in present post-fire vegetation studies, i.e. 30 and 120 years for Lac du Loup and Lago Perso, respectively. This probably results from the time required for Ericaceae to produce sufficient amount of seeds and biomass which could then be recorded in lake sediments. Another explanation of this lag between the expected and observed recovery time of ericaceous species might be the slow growth rate of *Vaccinium* mats, which can take decades to develop. This is supported by our precise identification of macroremains that indicate that ericaceous species were mostly *V. myrtillus* L. and *V. uliginosum* L. [27]. Variability in the severity of historical fires may also explain the variability in the response of vegetation studied here, as this has been shown to be an important consideration in boreal ecosystems [34], [35]. Further analyses of charcoal series should therefore attempt to reconstruct fire severity or fire size [36].

### A plant-fire functional interaction

The current pattern of land-use abandonment that characterizes European alpine areas results in the build-up of woody fuel and alters forest dynamics in such a way that *Larix decidua* is generally initially promoted [37], [38] followed by *Pinus cembra* later in the succession [39], [40]. Consequently, since our results underline the functional role of *Pinus cembra* as the main wildfire fuel, fire risk increases following land abandonment. Land abandonment also leads to a landscape-scale increase in the degree of connectivity between forests [41]. This changing pattern of vegetation cover, increasing stand-fuel and landscape connectivity, and global warming scenarios forecasting an increase in the frequency of droughts in southern Europe [10], [11], may result in an increased risk of fire during the 21st century. In subalpine ecosystems during the Holocene, fire promoted a typical pattern of secondary succession (Figure 3) as predicted by the Eggler's *initial floristic model* [42]. According to this model, although all species are present after a disturbance, species having functional life traits conferring a capacity to rapidly re-sprout and generate new growth (*Betula*, Ericaceae, herbs) are favored first, followed later by species that can withstand shading during their early growth and display other traits such as irregular seed production, and late fertility. In this secondary succession of subalpine ecosystems, the key functional species is *Pinus cembra*. However, this pine would be secondarily impacted by fire if the mean fire return interval were to be <150 years (Figure 3): indeed, the accumulation rate of *Pinus cembra* decreases during the first 150 years after a fire (Table 1). An increase in fire frequency, with a number of contiguous fires occurring with intervals <150 years would clearly threaten the *Pinus cembra* cover and would probably result in overcoming the resilience threshold of the present forested ecosystem, and so promote the development of herbs and shrubs. This scenario was probably recorded approximately 6700 years ago around Lac du Thyl [23], which is situated in the same valley as Lac du Loup. However, such an extirpation event never occurred at sites where the mean fire return interval failed to fall below 150 years; such as at Lac du Lait [43], Lac du Loup [27], and Lago Perso [44].

Long-term dynamics are apparent in systems in which the most prevalent species later in succession are the functional drivers of ecosystem disturbance (i.e. by providing fuel for fires). This system functions only if the fire occurrence is mainly driven by external factors such as climate, rather than by more intrinsic biological factors. To be resilient, an ecosystem needs a mean fire interval longer than the minimum time required to reach a late successional state, which is at least 150 years in the ecosystem studied here.

### Conclusion

Present-day mixed *Pinus cembra*-*Larix decidua* woodlands have formed natural subalpine ecosystems in our study area for at least 8000 years as a result of complex interactions and processes, including a fire return interval of ca. 300 years [27], [45] and the presence of *Pinus cembra* biomass which drives the fire frequency (present study). We do not rule out the role of other disturbances, e.g. avalanches, rock-falls, up-rooting of trees, or outbreaks of insects such as *Zeiraphera diniana*, that also affect subalpine forest dynamics and its landscape pattern [46]. Any alteration of the natural interactions between climate, disturbances, and human practices would most probably threaten the stable relationships between vegetation and fire that have been observed in the past. Our study suggests that an increased occurrence of fire would probably lead to a decline in the abundance of *Pinus cembra* to the benefit of *Larix decidua*, if the intervals between fires were less than 150 years. *Larix decidua* appears to be more resilient to fire and to have population dynamics that are not determined by fire alone, but by a complex of interactions among various disturbances.

## Materials and Methods

### Study sites

Two subalpine, north-facing lakes situated in the dry western Alps - Lac du Loup (45°11′14″N; 6°32′16″E, France) and Lago Perso (44°54′21″N; 6°47′50″E, Italy) - were cored using a “Russian” corer and a Kajak-Brinkurst sampler (details given in [27]). Lac du Loup (1400 m^2^, 0.65 km^2^ watershed) is situated at 2035 m a.s.l. within the municipality of Orelle in the Maurienne Valley, France (Figure S1), whereas Lago Perso (408 m^2^, 0.27 km^2^ watershed) is at 2000 m a.s.l. within the municipality of Cesana Torinese in the Susa Valley, Italy (Figure S1).

Local vegetation around the two lakes is a mixed stands of European larch (*Larix decidua* Mill.) and arolla pine (*Pinus cembra* L.) with scattered mountain pines (*Pinus mugo* subsp. *uncinata* (DC.) Domin.) and Norway spruce (*Picea abies* (L.) H. Karst.). The woody understoreys are characterized by Ericaceae (*Vaccinium myrtillus* L., *V. vitis-idaea* L., *V. uliginosum* L., *Rhododendron ferrugineum* L., *Arctostaphyllos uva-ursi* (L.) Spreng.) and *Juniperus sibirica* Lodd. Ex Burgsd with scattered mats of *Empetrum nigrum* subsp. *hermaphroditum* (Hagerup) Böcher. Pastures dominated by short grasses, Poaceae and Cyperaceae, occur around the lakes as grazed woodlands or treed grasslands. They are presently grazed by cattle (Lago Perso) or sheep (Lac du Loup) annually each summer.

The continental-type climate is characterized at Lago Perso by ∼880 mmyear^−1^ of mean precipitation as rain and snow [40]. More precise climate data at St Michel-de-Maurienne (1360 m a.s.l., ∼2 km from Lac du Loup) indicate a mean precipitation of 947±184 mm.year^−1^ and a mean annual temperature of 7.1±0.6°C (January: −0.2±2.2°C; July: 15.5±1.6°C). Bedrocks are composed of permo-carboniferous schists and sandstones (Lac du Loup) or from calc-schists (Lago Perso, [40]) with acidic soils and podzols occurring under mature forests.

### Plant macroremains analysis, dating, and age-depth modeling

Plant macroremains (needles, leaves, seeds, cones, pollen sacs, etc.) were retrieved at high resolution (1 cm) from sediment cores by soaking. The extraction was carried out by water sieving, after which macroremains were identified and counted. The detailed macroremains diagrams have been published elsewhere [27], [44]. In the present study we focus solely on the main functional forest taxa (*Larix decidua*, *Pinus cembra*, *Betula* sp., total Ericaceae, and total herbs) whose abundance is expressed in influx (cm^−2^.yr^−1^) using solid age-depth models based on a total of 21 calibrated ^14^C datings of plant macroremains and ^210^Pb measurements (details given in [27], [44]). Calibrated ages before present are denoted as ‘cal BP’.

### Fire reconstruction

To reconstruct fire history, the surface areas of sedimentary charcoal were tallied continuously at high resolution (every centimeter) and the Charcoal Accumulation Rate (CHAR mm^2^.cm^−2^.yr^−1^) was time-analyzed to reconstruct the stand-to-local fire history of the two lakes. CHAR series are composed of two subpopulations of charcoal: first, the CHAR-background, representing the variations in overall charcoal production, sedimentation, mixing and sampling; and second, CHAR-peaks exceeding the CHAR-background that are assumed to be related to fires [47], [48]. Thresholds applied to the CHAR-peak component allowed the detection of any relevant peaks that exceeded the background noise within the record, and which was assumed to be related to an occurrence of fire. All analyses were performed using CharAnalysis 1.0 software [3].

### Superposed epoch analysis (SEA)

We used SEA to examine the behavior of macroremain influxes before and after fires. Macroremain influxes are primarily interpolated at a constant time-step of 15 years. Because the abundance of plant macroremains displays a high magnitude of fluctuation (Figure 1C and 1D), selected influxes were Log-transformed to reduce the weight of extreme values ([49], Figure 1E and 1F), such as:



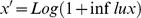



To remove the autocorrelation in the macroremain influx series we deconstructed the time series into high- (Figure 1G,H) and low-frequency trend (Figure 1I, J) and residual components (Figure 1K,L) using LOESS [50]. The high-frequency trend in macroremain series is found by LOESS smoothing with a 500 years' time window, here supposed to be large enough to filter high-frequency component. The high-frequency trend is removed, and the remainder smoothed to find low-frequency trend. Macroremain influx residuals from the high plus low-frequency trend fit are used as independent samples to perform SEA (Figure 1K and 1L). To test the response of detrended macroremain influx series to multiple fires, SEA involves sorting data following time windows dependent on a key-date (here fire events) to synchronize and compare the means of those time windows. This method involves a simple compositing (averaging) of different signals to detect deviations from the background rate [16], [17]. For each 15-year fire event, a 240-year window was selected with 90 years before and 150 after the fire event (17 data points). A window of this size should be large enough to detect any significant response to fire events. Monte Carlo resampling techniques, involving randomly picking fire events from the chronology (1000 repetitions) was used to estimate the 95 and 99% confidence intervals (CI) around the averaged macroremain influx values [51]. To highlight the response pattern of taxa to fire, SEA composites were displayed as anomalies using the following transformation:



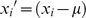



where *x_i_′* is the transformed value of the *i^th^* sample, and *µ* is the mean *x_i_* value (Figure 1K,L). Wilcoxon Mann-Whitney non-parametric tests were used to compare pre- and post-fire average macroremain influxes following different time windows of 60, 90 and 150 years [20] to decipher the lagged response of averaged macroremain influx to fires.

Species-specific SEA scores were then used to produce a comprehensive pattern of forest dynamics linked to fire. After a min/max rescaling of SEA scores by species:



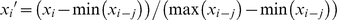



a LOWESS curve fitting was used to highlight temporal trajectories of transformed macroremain influxes. The time resolution of the SEA is 15 years because of the temporal resolution of interpolated macroremains series and the uncertainties of ^14^C chronologies inferred from age/depth models (Lac du Loup, [27]; Lago Perso, [44]).

## Supporting Information

Figure S1 Location map of studied sites
(5.41 MB TIF)
