# Supplementary figures and images for: Fire, Fuel Composition and Resilience Threshold in Subalpine Ecosystem
Source: PLoS One. 2010 Aug 30;5(8):e12480. doi: 10.1371/journal.pone.0012480 (PMC2930012; doi:10.1371/journal.pone.0012480)

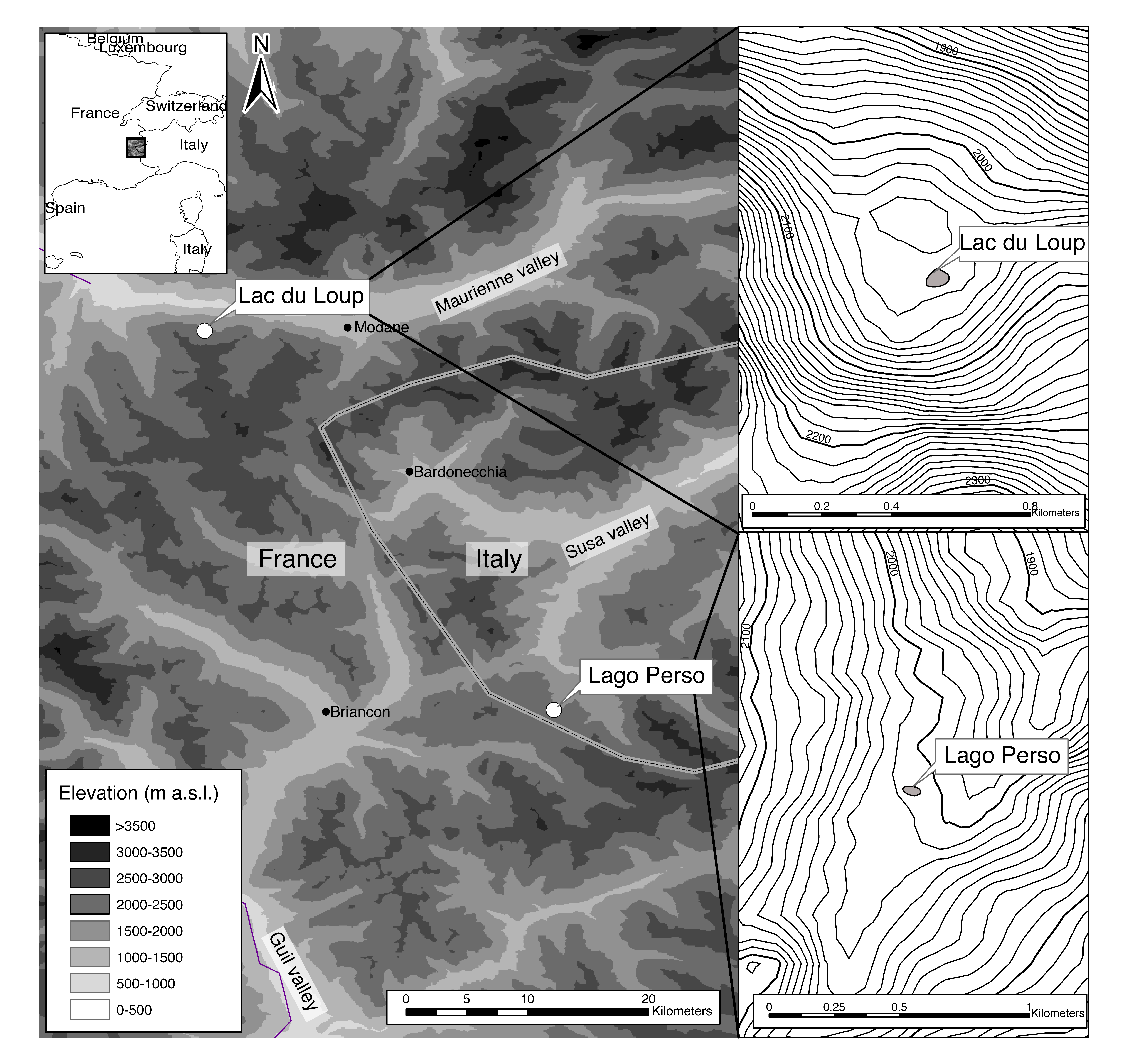

Supplement: Figure S1 — Location map of studied sites (5.41 MB TIF) [file pone.0012480.s001.tif]
